# Learning influences host choice in tsetse

**DOI:** 10.1098/rsbl.2006.0578

**Published:** 2006-12-19

**Authors:** Jérémy Bouyer, Mathieu Pruvot, Zacharia Bengaly, Patrick M Guerin, Renaud Lancelot

**Affiliations:** 1 Centre de coopération internationale en recherche agronomique pour le développement (CIRAD), Département d'élevage et de médecine vétérinaire34398 Montpellier Cedex 5, France; 2 Centre international de recherche-développement sur l'élevage en zone subhumide (CIRDES)BP454, Bobo-Dioulasso, Burkina Faso; 3 University of Neuchâtel, Institute of ZoologyRue Emile-Argand 11, 2009 Neuchâtel, Switzerland

**Keywords:** tsetse flies, trypanosomosis, feeding preference, learning

## Abstract

A learning capacity for feeding is described in many insect species including vectors of diseases, but has never been reported in tsetse flies (Diptera, Glossinidae), the cyclic vectors of human (sleeping sickness) and animal trypanosomoses in Africa. Repeated feeding on the same host species by a disease vector is likely to increase the within-species disease-transmission risk, but to decrease it between species.

An experiment with cattle and reptiles in a stable provides evidence that the species of host selected for the second blood meal in tsetse flies depends on the host encountered for the first blood meal when the between-meal interval is 2 days. This preference disappears when the between-meal interval is extended to 3 days. The energetic advantages of this acquired preference and its importance in trypanosomoses epidemiology are discussed.

## 1. Introduction

In insects, an acquired feeding preference can overcome variations in the quality and distribution of food resources, as described for honeybees (*Apis mellifera*), other hymenoptera and vectors of diseases, with important epidemiological implications for the latter (Thorpe & Jones 1937; Lewis & Tuminson 1988; Bicker & Hähnlein 1994). For example, mosquitoes tend to return to the same villages, houses, host species and oviposition sites (McCall & Kelly 2002).

The biological vectors of animal and human trypanosomoses in West Africa are tsetse flies of the *palpalis* group (Genus *Glossina*, subgenus *Nemorhina*) living in riparian vegetation (Challier & Gouteux 1980; Bouyer *et al*. 2005*b*, 2006), that feed on hosts including reptiles, ruminants and humans (Weitz 1963). Within the same tsetse species, between-population differences are observed in the hosts fed on, depending on host availability (de La Rocque *et al*. 2005). Preliminary experiments suggested that tsetse flies might possess a learning capacity (Bouyer *et al*. 2005*a*); this paper uniquely demonstrates that a tsetse vector's first host encounter can strongly influence host choice on subsequent feeding cycles, with the magnitude of this effect being diminished by starvation.

## 2. Material and methods

On-station experiments were made in a stable (10.4×4.0×2.0 m high), isolated by mosquito netting at CIRDES, Burkina Faso, with cohorts (125 flies per cohort) of teneral male *Glossina palpalis gambiensis* (referred to hereafter as ‘flies’) drawn from a laboratory population (size approximately 100 000). Flies were marked the day after emergence (with acrylic paint on the pronotum) with different colours according to the cohort and rotations between cohorts. On day 2, separate cohorts were exposed to their first host (no choice situation) consisting of either caged (mesh size 2.5×5.0 cm) reptiles (R) or a tethered bovid (C) placed in the stable before the flies (see table 1 for details of the experimental design): two monitor lizards, *Varanus niloticus* coded *V. n*. 1 (190 cm long, 3 kg) and *V. n*. 2 (100 cm long, 2 kg); a crocodile, *Crocodilus niloticus* coded *C. n*. (115 cm long, 15 kg); a 5-year-old 134 kg female *Bos Taurus* coded *B. t*., Baoulé breed; a 4-year-old 155 kg female *Bos indicus* coded *B. i*. 1, local breed; and finally a 6-year-old 336 kg male *B. indicus* coded *B. i*. 2, local breed. In four trials of nine, two monitor lizards were put in the same cage with two other lizards to provide a total host bait mass closer to that of one cow. Flies were left for 2 h (between 8 and 12.00 a.m.) and captured before host removal (two persons with protective clothing for 30 min). Engorged flies (average 88 per cohort) were released 2 (seven repetitions, minimal between-meal lag (BML) observed in nature) or 3 days later (two repetitions) with a similar number of teneral flies (no feeding history, eight repetitions) into the same stable but now holding both hosts (R+C, choice situation). Replete flies (average 63 per cohort) were caught for dissection to determine the origin of the blood in the crop. From well-established knowledge of tsetse physiology, the blood meal is evacuated from the crop and anterior midgut within 3 h, thus excluding confusion of blood types between hosts of two successive meals. Thin smears were made of the blood meals and stained (10% Giemsa) for host species determination by microscope examination of erythrocytes: oval and nucleated for reptiles, round and anucleated for cattle.

The analysed response of flies was the proportion *π* that fed on cattle (choice situation). Our aim was to test the hypothesis that previous experience feeding on a host increases the probability a tsetse will feed on the same species on a second feed. The explanatory variables were (i) first host species (bovid, reptile or none for teneral flies) and (ii) the BML (2 or 3 days). Categories were (i) starvation, (ii) first meal on a bovid and BML of 2 days, (iii) first meal on reptile and BML of 2 days, (iv) first meal on a bovid and BML of 3 days, and (v) first meal on reptile and BML of 3 days. Preliminary analyses showed that the variance of the response was greater than the expected binomial variance. To account for this, a beta-binomial logistic regression model was applied (Griffiths 1973): a within-batch correlation coefficient *ϕ* was estimated in addition to the coefficients of the fixed effects. Wald tests were used to test linear combinations of the coefficients under various null hypotheses. Statistical analyses were made with R software (R Foundation for Statistical Computing, Vienna; http://www.R-project.org) and the R package aod (v. 1.1-10; http://cran.r-project.org/).

Probabilities $\hat{\pi }$ were fitted for each category of the explanatory variable. To facilitate their interpretation, relative risks (RRs) were computed as follows: ${\hat{RR}}_{i,j}={\hat{\pi }}_{i}/{\hat{\pi }}_{j}$, where ${\hat{\pi }}_{i}$ was the fitted probability of a fly choosing a bovid or a reptile for its second blood meal, given its feeding history, and ${\hat{\pi }}_{j}(i\neq j)$ was the fitted probability to be compared with. Model coefficients were regarded as the realization of a multivariate Gaussian distribution, with the coefficients as the mean, and their variance–covariance matrix as the variance. Ten thousand samples drawn from this distribution were used to compute the corresponding RRs. The empirical 0.025 and 0.975 quantiles of the simulated values were used to compute their 95% confidence intervals.

## 3. Results

The engorgement rates of teneral flies were similar on reptiles (0.63, s.d. 0.16) and bovids (0.70, s.d. 0.15), and did not increase when both were presented together (0.62, s.d. 0.16). The engorgement rates of already fed flies on both hosts were slightly higher (0.73, s.d. 0.18), but did not change with the origin of the first host nor the BML (*p*>0.05).

The estimated within-batch correlation coefficient was $\hat{\phi }=0.06$ (*z*=2.85, *P*(>*z*) =0.002), thus confirming the need to use a statistical model accounting for this correlation. Teneral flies preferred to feed on cattle: $\hat{\pi }$=0.85 (0.79; 0.92) (95% confidence interval), a preference also observed for flies previously fed on cattle at a BML of 2 days ($\hat{\pi }$=0.94 (0.89; 0.99)). Flies previously fed on reptiles preferred reptiles ($\hat{\pi }$=0.44 (0.34; 0.55)). The fitted probabilities for the latter were significantly different from the former (d.f.=1, 20; *P*=0.03 for cattle and *P*=2×10^−8^ for reptiles). Observed data and fitted probabilities are shown (figure 1). When BML was 3 days, the preference for cattle was similar independent of the first meal source and not significantly different from teneral flies (first meal=reptile: d.f. =1, 20, *P*=0.74; first meal=cattle, d.f. =1, 17, *P*=0.33); model outputs were compatible with a common probability for teneral flies and flies previously fed on cattle or reptiles (d.f.=2, 20; *P*=0.59).

RRs are displayed in table 2 for a BML of 2 days (none were different from 1 when the BML was 3 days). For example, for tsetse that had taken their first meal from cattle 2 days earlier, the RR of feeding on cattle versus reptile for their second meal was 15.94, whereas for tsetse that had taken their first meal from cattle 2 days earlier, the RR of feeding on cattle versus reptile for their second meal was 2.1 in comparison with tsetse that had taken their first meal from reptile(s). The value of each RR was compatible with the assumption that the feeding history influences the choice of host for the second blood meal in the expected direction: under these experimental conditions, flies with a first blood meal on cattle preferred cattle over reptiles for their second blood meal while those feeding first on reptiles preferred reptiles for their second blood meal. All RR values but 2 (second column, fourth line in table 2*a* and second column, second line in table 2*b*) were significantly different from 1.

## 4. Discussion

These experiments show how the first host selected by *G. palpalis gambiensis* for a blood meal can influence the host selected for the second meal when the BML is 2 days. This acquired feeding preference seemed to be lost through starvation, but data are limited in terms of replication and other explanations are possible. However, it argues for a strong role of environmental, in addition to genetic, determinants of host choice. The preference of teneral flies for cattle might be related to host size, but specific attraction could also be involved. The behaviour of our laboratory-reared flies may be different from those in the wild. However, earlier observations indicated that *G. palpalis gambiensis* that had fed on goats in captivity still preferred this host after their release in nature (Challier 1973).

High seasonal and geographical variations in the availability of hosts occur in *G. palpalis gambiensis* habitats. To manage host transience, a learning ability would allow flies to focus on available species (McCall & Kelly 2002). This is crucial for tsetse populations that cannot survive feeding-related mortality greater than 3% (Schofield & Torr 2002). Loss of host preference with starvation could be a manifestation of opportunistic feeding behaviour: a fly waiting for cattle at water would be better feeding on a suid or reptile than starve to death.

By reducing the probability of between-species trypanosome transmission, learning probably influences host–parasite coevolution and parasite pathogenicity (Combes 2001), but it is difficult to forecast in which direction: whereas within-cattle trypanosome transmission reduced pathogenicity (Van Den Bossche *et al*. 2003), species-specific malarial parasites were found to be more virulent than generalists (Garamszegi 2006). Further work is needed, both on fly-feeding behaviour and parasite genetic variability in cattle and other hosts to determine whether host specialization generates higher or lower virulence.

Learning in tsetse flies may also play a role in the epidemiology of sleeping sickness. Selective host choice might increase the basic reproductive rate of the disease and the vectorial capacity of flies (Dye & Hasibeder 1986), and the phenomenon might be amplified by a greater feeding success on morbid hosts (Baylis & Nambiro 1993). Nevertheless, the acquired preference is not exclusive and spatial encroachment between man and animal reservoirs probably favours between-species transmission (Sané *et al*. 2000).

Learning in tsetse populations may contribute to the biological and epidemiological isolation of subpopulations and consequently restrict gene flow. Genetic clusters occur in close tsetse fly populations (Solano *et al*. 2000), despite their dispersal capacities (Bouyer *et al*. 2007).

## Figures and Tables

**Figure 1:**
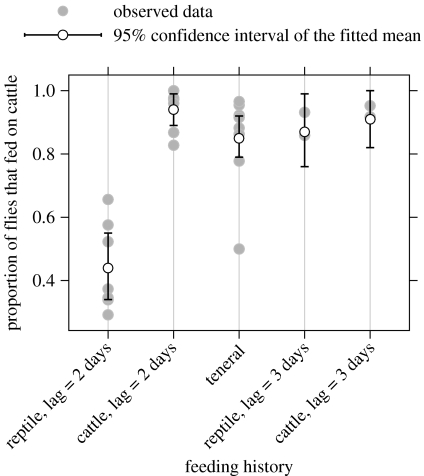
Observed and fitted probabilities (beta-binomial regression model) that the tsetse fly *Glossina palpalis gambiensis* feeds on a bovid given its feeding history (teneral fly, or previous meal on a reptile or bovid with a lag of 2 and 3 days).

**Table 1:** Host exposed during the experiments and inter-meal periods (*V. n*., *Varanus niloticus*; *C. n*., *Crocodilus niloticus*; *B. i*., *Bos indicus* and *B. t*., *Bos Taurus*).

date of first release	13 Apr 2005	19 Oct 2005	25 Nov 2005	8 Dec 2005	14 Feb 2002	18 Jul 2003	4 Jul 2006	2 Apr 2005	18 Apr 2005
reptile (first meal)	*V. n*. 1	*V. n*. 1+*V. n*. 2	*V. n*. 1+*V. n*. 2	*V. n*. 1+*V. n*. 2	*V. n*. 1	*V. n*. 1	*V. n. 2*	*C. n*.	*V. n*. 1+*V. n*. 2
bovid (first meal)	*B. i*. 1	*B. t*.	*B. i*. 2	*B. i*. 2	*B. t*.	*B. t*.	*B. i*. 1	*B. i*. 1	*B. t*.
reptile (second meal)	*V. n*. 1	*V. n*. 1+*V. n*. 2	*V. n*. 1+*V. n*. 2	*V. n*. 1+*V. n*. 2	*V. n*. 1	*V. n*. 1	*V. n*. 1	*C. n*.	*V. n*. 1+*V. n*. 2
bovid (second meal)	*B. i*. 1	*B. t*.	*B. i*. 2	*B. i*. 2	*B. t*.	*B. t*.	*B. i*. 2	*B. i*. 1	*B. t*.
inter-meal period (days)	2	2	2	2	2	2	2	3	3

**Table 2:** (*a*, *b*) Relative risks ${\hat{RR}}_{i,j}={\hat{\pi }}_{i}/{\hat{\pi }}_{j}$ for the tsetse fly *Glossina palpalis gambiensis* of feeding on (*a*) a bovid or (*b*) a reptile for its second blood meal (${\hat{\pi }}_{i}$, between-meal lag of 2 days) given its feeding history, with various reference situations (${\hat{\pi }}_{j}$). (Probabilities were estimated with a beta-binomial regression model. For example, for tsetse that had taken their first meal from cattle 2 days earlier, the RR of feeding on cattle versus reptile for their second meal was 15.94, whereas for tsetse that had taken their first meal from cattle 2 days earlier, the RR of feeding on cattle versus reptile for their second meal was 2.1 in comparison to tsetse that had taken their first meal from reptile(s). M, M_2_, M_1_: blood meal, the index giving its rank in case of successive meals; R: reptile; B: bovid and T: teneral fly.)

${\hat{\pi }}_{j}$	${\hat{\pi }}_{i}$	

	P(M_2_=B|M_1_=B)	P(M_2_=B|M_1_=R)
(*a*)		
P(M_2_=B|M_1_=B)	—	0.47 (0.36, 0.60)
P(M_2_=B|M_1_=R)	2.12 (1.67, 2.77)	—
P(M_2_=R|M_1_=B)	15.94 (6.80, 36.88)	7.53 (3.32, 17.12)
P(M_2_=R|M_1_=R)	1.69 (1.40, 2.11)	0.80 (0.51, 1.25)
P(M=B|T)	1.10 (1.01, 1.22)	0.52 (0.40, 0.66)

	P(M_2_=R|M_1_=B)	P(M_2_=R|M_1_=R)
(*b*)		
P(M_2_=B|M_1_=B)	0.06 (0.03, 0.15)	0.59 (0.47, 0.71)
P(M_2_=B|M_1_=R)	0.13 (0.06, 0.30)	1.25 (0.80, 1.94)
P(M_2_=R|M_1_=B)	—	9.40 (4.22, 21.34)
P(M_2_=R|M_1_=R)	0.11 (0.05, 0.24)	—
P(M=R|T)	0.40 (0.17, 0.96)	3.02 (1.80, 5.06)
